# TCR Repertoire Analysis Unveils the Link Between Kawasaki Disease and Viral Infection

**DOI:** 10.3390/biomedicines14030574

**Published:** 2026-03-03

**Authors:** Zhimi Geng, Wei Zhou, Zhihao Fang, Yihua Jin, Guoqiang Qi, Lin Zhao, Chunhong Xie, Yujia Wang, Fangqi Gong

**Affiliations:** 1 Department of Cardiology, Children’s Hospital, Zhejiang University School of Medicine, National Clinical Research Center for Children and Adolescents’ Health and Diseases, Hangzhou 310052, China; 2 Pediatric Cardiovascular Diseases Laboratory, Children’s Hospital, Zhejiang University School of Medicine, National Clinical Research Center for Children and Adolescents’ Health and Diseases, Hangzhou 310052, China; 3 Department of Nephrology, Children’s Hospital, Zhejiang University School of Medicine, National Clinical Research Center for Children and Adolescents’ Health and Diseases, Hangzhou 310052, China; 4 Department of Data and Information, Children’s Hospital, Zhejiang University School of Medicine, National Clinical Research Center for Children and Adolescents’ Health and Diseases, Hangzhou 310052, China; 5 Department of Cardiology, Hangzhou Traditional Chinese Medicine Hospital Affiliated to Zhejiang Chinese Medical University, Hangzhou 310007, China

**Keywords:** Kawasaki disease, viral infection, TCR repertoire, VJ gene segments, V-J pairs

## Abstract

**Background**: Kawasaki disease (KD) is a systemic vasculitis of unknown origin, though recent evidence implicates viral pathogens in its pathogenesis. Given the central role of T cell receptors (TCRs) in antigen recognition and immune response, this study investigated the association between KD and viral infection through comparative analysis of TCR repertoires. **Methods**: TCR repertoires from KD patients, healthy children, and individuals with viral infections were comparatively analyzed. TCR diversity and V(D)J usage were assessed using Shannon’s entropy, the Mann–Whitney U test, and Fisher’s exact test. Positional motif enrichment analysis within CDR3 regions was performed based on paratope hotspot classification. **Results**: Relatively reduced TCR clonal abundance and diversity were observed in KD patients compared to healthy controls. While substantial overlap in VJ gene segment usage was detected between KD and cytomegalovirus (CMV) infection, limited overlap in clonal TCRαβ chains was found between KD and viral infection groups. A predominant TCR combination, TRAV14DV4-J13-TRBV20-1-J2-5, enriched with characteristic amino acid motifs (EET, YNE, LAG, GQG, and AYE), was frequently identified in KD. **Conclusions**: These observations suggest potential differences in TCR repertoire features between KD patients and both healthy and virus-infected groups. However, the relationship between KD pathogenesis and the viruses examined requires further investigation with larger cohorts.

## 1. Introduction

Kawasaki disease (KD) is a pediatric disorder commonly seen in children younger than 5 years old, in which systemic vasculitis is the main lesion, manifested by multiple clinical manifestations such as fever and rash. KD presently stands as the preeminent etiology of acquired cardiac pathology in children due to its susceptibility to coronary artery damage [1]. Although the cause of KD remains controversial, the prevailing speculation is that KD is caused by infectious agents that infect genetically susceptible individuals and trigger inflammatory mechanisms, which target cardiovascular organs [2]. The apparent seasonality, clinical manifestations and laboratory findings of KD significantly overlap with most infections [3,4], indicating that infectious triggers may be involved in the pathogenesis of the inflammatory process in KD. Among the infectious factors, viruses were mainly explored, including cytomegalovirus (CMV), herpes simplex virus type 2 (HSV-2), Epstein–Barr virus (EBV), influenza A (IFA) and severe acute respiratory syndrome coronavirus 2 (SARS-CoV-2), which causes pediatric multisystemic inflammatory syndrome (MIS-C) [5,6,7,8,9,10]. Even if the above studies suggest that KD is a virus-related clinical syndrome, the evidence remains poor.

There is increasing evidence that abnormal adaptive immune responses are involved in the pathogenesis of KD [2,11]. Concurrently, the adaptive immune system, particularly the actions of T lymphocytes, is pivotal in the resolution of viral pathogens [12]. T cell receptor (TCR) and major histocompatibility complex (MHC) molecules, expressed on the surface of T cells, recognize pathogen-associated epitopes via interactions with peptide-MHC (pMHC) and respond by activation. In children with idiopathic nephrotic syndrome, when the T cell is activated, it proliferates rapidly, and a mass of T cells bearing identical T cell receptors is generated [13]. TCR is generated by the somatic rearrangement of variable (V), diversity (D), and joining (J) gene segments at the early stage of T cell development, known as V(D)J recombination [14]. Complementary determining region 3 (CDR3), demarcating the succinct expanse bridging the VD and VJ junctions, emerges as the quintessentially variable segment and is predominantly scrutinized for its cardinal role in antigen identification [15].

Here, we asked whether KD is a virus-related clinical syndrome and whether TCR repertoires are selectively skewed in children suffering from KD. The TCR profile of KD patients was compared with children with viruses, including CMV, HSV-2, EBV, IFA and SARS-CoV-2, in this study. Similarities and differences in TCR repertoires in the peripheral blood of KD and virus-infected patients will be revealed, which will illuminate fresh perspectives on the function of viruses in KD in terms of T cell activation.

## 2. Materials and Methods

### 2.1. Samples

On the one hand, we collected clean blood samples from three Kawasaki disease patients who were on admission without any treatment, along with samples from an equal number of healthy children, to perform single-cell V(D)J sequencing analysis. Eligibility of KD subjects was ascertained based on the benchmarks proffered by the American Heart Association [16]. Utilizing bidimensional echocardiography, the investigative team appraised the presence of any cardiac functional anomalies or coronary arterial perturbations during the acute and subsiding stages of the ailment. Responding favorably to intravenous immunoglobulin therapeutics, none of the patients progressed to coronary arterial complications at the three-month post-disease inception. The healthy cohort displayed an absence of pyrexia, infectious conditions, or recent immunizations. All experimental procedures were conducted in accordance with relevant guidelines and regulations and were approved by the Institutional Review Board of the Children’s Hospital, Zhejiang University School of Medicine (IRB approval number: 2021-IRB-320). As this research involved retrospective analysis of medical records and specimens, the IRB granted a waiver of informed consent. A detailed compendium of the clinical data for the subjects has been collated in Supplementary Table S1. On the other hand, a publicly available V(D)J sequence dataset on the Peripheral Blood Mononuclear Cells (PBMCs) of 6 KD patients before treatment and 3 healthy children under the age of 5 was fetched from the GEO database (GSE168732). In total, a sum of 9 KD patients and 6 healthy children diagnosed with complete Kawasaki disease from our own data and the public database were included. In addition, single-cell TCR data of virus-infected samples were obtained from VDJdb (https://vdjdb.cdr3.net/), including CMV, HSV-2, EBV, IFA and SARS-CoV-2. Only samples with a cell number greater than 100 were used for subsequent analysis.

### 2.2. Single-Cell Preparation and Sequencing

For every participant yielding to the single-cell V(D)J sequencing assay, 2 milliliters of venous blood was collected at the acute stage before intravenous immunoglobulin and corticosteroid treatment. Following extraction, the blood was expeditiously processed within a quadruple-hour window. Peripheral Blood Mononuclear Cells (PBMCs) were segregated via density gradient centrifugation, employing Ficoll-Paque as the medium of choice. A meticulously calculated aliquot of the cellular mixture was prepared, designed to encompass an approximate aggregate of 10,000 cells per specimen. The intricate process of single-cell apprehension and subsequent library synthesis was executed by employing the Chromium Next GEM Single Cell V(D)J Reagent Kit version 3, produced by 10× Genomics (Pleasanton, CA, USA), adhering stringently to the protocols prescribed by the manufacturer. In summary, the cellular blend, replete with barcoded gel beads and partitioning oil, was dispensed onto the 10× Genomics Chromium Chip, thereby creating single-cell Gel Beads-encased Emulsions. The ensnared cells were then lysed, with the ensuing transcripts being barcoded via reverse transcription within the confines of each solitary Gel Bead-encased Emulsion. The libraries, showcasing each cell’s 5′ end and V(D)J regions, were then sequenced using Illumina’s Novaseq 6000 platform, deploying a sequencing strategy of 150-base-pair paired-end reads, with quality control metrics provided in a Table S2 in the Supplementary Materials.

### 2.3. Single-Cell 5′V(D)J Data Processing

Reconstruction of TCR repertoires was conducted using the Cell Ranger VDJ pipeline (version 3.1.0). All parameters were kept as default (https://www.10xgenomics.com/support/software/cell-ranger/latest/analysis/running-pipelines/cr-5p-vdj, accessed on 28 February 2026). The assembly process for each cell barcode includes: (1) building a de Bruijn graph of observed 20-mers; (2) extending k-mers to generate putative contigs; (3) assigning each UMI to a single contig based on read alignment scores; (4) outputting contigs supported by at least one UMI. We used the 10x Genomics provided by the V(D)J reference (refdata-cellranger-vdj-GRCh38-alts-ensembl-2.0.0) for alignment and annotation. Only productive, full-length contigs with annotated CDR3 sequences were retained for downstream analysis.

### 2.4. Statistical Analysis

The distributions reflecting clonal proliferation were deciphered through the application of resampling methodologies, a strategy implemented to rectify any disparities attributable to fluctuations in sequencing profundity. To quantify the clonal heterogeneity present within each specimen, the mathematical measure of Shannon’s entropy was utilized. The employment of the hypergeometric test facilitated the evaluation of the significance of shared V-J pairings amongst KD patients compared with a cohort of healthy juveniles. Discrepancies between the patient and control groups were scrutinized utilizing either the Mann–Whitney U test or Fisher’s exact test, adopting a threshold of significance demarcated by a *p*-value less than 0.05. The entirety of this statistical scrutiny was executed utilizing the R computational platform (version 3.5.1) [17]. Given the exploratory nature of this study, the reported *p*-values were not adjusted for multiple comparisons. All statistical inferences should be considered hypothesis-generating and require validation in future independent studies.

To infer T cell receptor antigen specificity, we utilized the Grouping of Lymphocyte Interactions by Paratope Hotspots (GLIPH) algorithm [18]. This method systematically clusters T cell receptor sequences based on shared global and local amino acid motifs within their complementarity-determining region 3 sequences. The resulting clusters were then rigorously analyzed for statistically significant enrichments of specific features, including shared variable gene usage, uniform CDR3 length, clonal expansion, and associated HLA alleles. These enrichments were integrated to calculate a specificity group score for each cluster, defining groups of T cell receptors with likely shared antigen recognition. The parameter settings used for the GLIPH analysis are provided in the Table S2.

### 2.5. Data Availability

The raw sequence data reported in this paper have been deposited in the Genome Sequence Archive (Genomics, Proteomics & Bioinformatics 2025) in the National Genomics Data Center (Nucleic Acids Res 2025), China National Center for Bioinformation/Beijing Institute of Genomics, Chinese Academy of Sciences (GSA-Human: HRA015796), which are publicly accessible at https://ngdc.cncb.ac.cn/gsa-human (accessed on 28 February 2026) [19,20].

## 3. Results

### 3.1. KD Patients Showed Relatively Low TCR Abundance and Diversity

**Figure 1:**
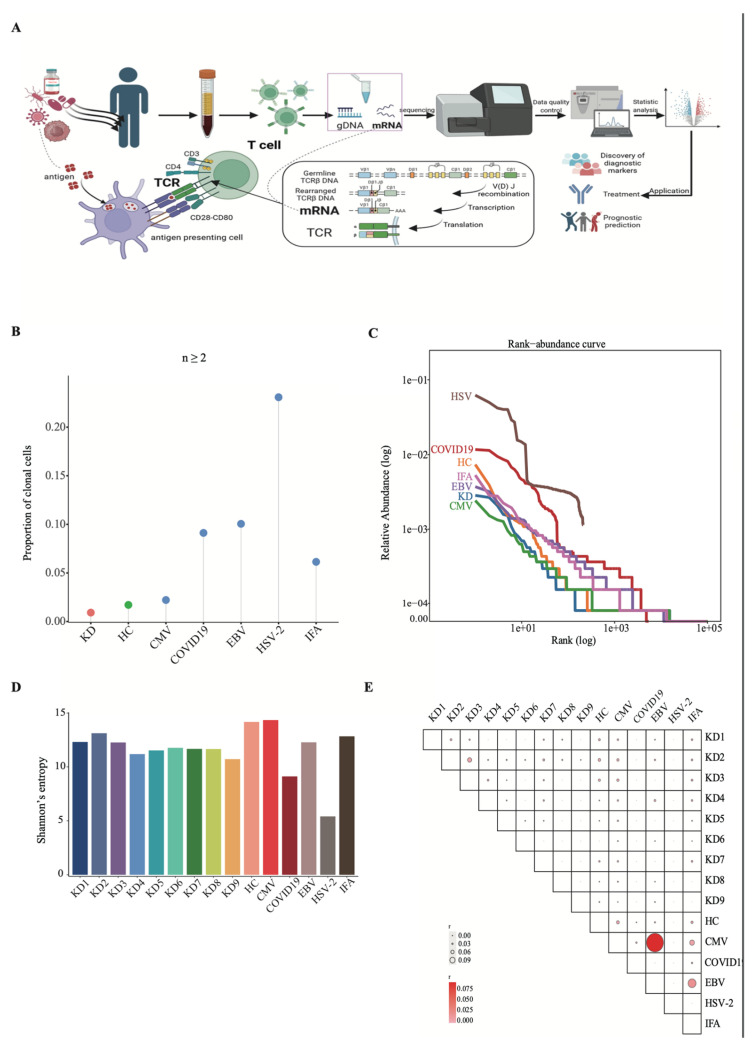
Characterization of TCRαβ chains in KD. (**A**) Schematic overview of the study design. Blood samples from KD patients (n = 9) and healthy children (HC, n = 6) were analyzed by single-cell TCR V(D)J sequencing. (**B**) Proportion of expanded TCRαβ clones (clonal frequency ≥ 2) in indicated groups. (**C**) Rank abundance distribution of TCRαβ clones across groups. Statistical testing was not performed due to variations in sequencing depth. (**D**) Shannon entropy of TCRαβ repertoires. Data are presented as mean ± SD; no statistical testing was performed, as this metric is derived from clonal abundance. (**E**) Clonal similarity matrix (Jaccard index) of TCRαβ sequences between indicated groups. Data are presented descriptively.

Blood samples of KD patients and healthy children were obtained to perform single-cell V(D)J sequencing, and V(D)J sequence datasets were obtained. TCR profile of 9 KD samples, six samples of healthy children and viral infection were analyzed (Figure 1A). T cells recognize pathogens on the basis of the diversity of the TCR repertoire composed of α and β chains. Once activated, T cells produce large numbers of T cells with the same paired TCRαβ chains, namely cloned cells [21]. To explore TCR bias in KD patients, we conducted a comparative analysis of paired TCRαβ chains in KD patients, healthy children and viruses. The average proportion of clonal TCRαβ chains (n ≥ 2) was lower in KD patients than in healthy children and samples infected with the virus (*p* < 0.05, Fisher’s exact test) (Figure 1B). Rank abundance distribution estimator and Shannon entropy were used to analyze TCRαβ chains of KD patients, healthy children and virus-infected samples. Descriptively, HSV-2- and COVID-19-infected samples exhibited higher clonal abundance and lower diversity compared to KD samples (Figure 1C,D). These findings suggest that HSV and COVID-19 infection may elicit a more robust T-cell immune response in peripheral blood than in KD patients in the early acute phase, although validation in larger cohorts is needed. Regarding the similarity analysis of TCRαβ clonal sequences, limited overlap was observed between KD and virus-infected samples (Figure 1E). However, due to differences in sequencing depth, sample processing, and small sample size, we cannot definitively conclude the absence of shared clonotypes, warranting further validation with standardized methods and larger cohorts.

### 3.2. Distinct V and J Gene Segment Usage and V-J Pairing Patterns in KD Patients Compared to Healthy Controls

**Figure 2:**
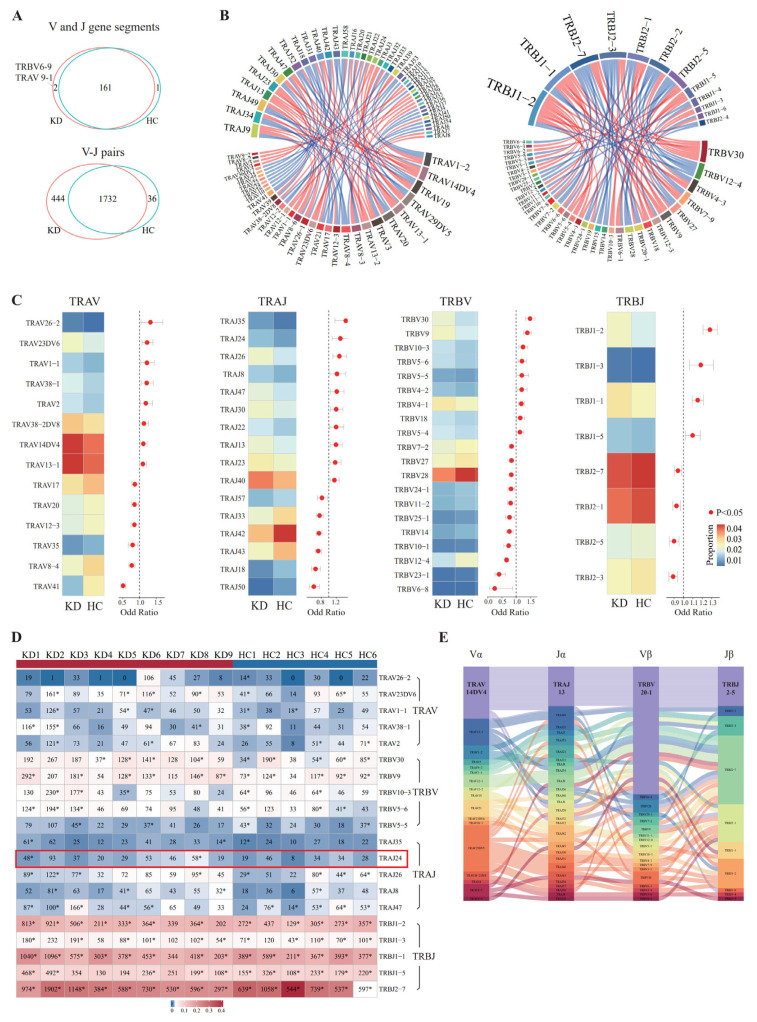
Comparison of VJ gene segments and V-J pairs between KD and healthy children. (**A**) Venn diagrams showing shared and unique VJ gene segments (upper) and V-J pairs (lower) between KD (n = 9) and HC (n = 6). * *p* < 0.05, hypergeometric test (uncorrected for multiple comparisons due to exploratory nature of the analysis). (**B**) Heatmap of V and J gene segments enriched in KD compared to HC. Color scale indicates relative frequency. Enrichment was assessed by Fisher’s exact test with Benjamini–Hochberg correction for multiple testing (adjusted *p* < 0.05 considered significant; full statistics provided in Supplementary Table S1). (**C**) Clonal frequency of the top five increased V/J segments in KD and HC. Asterisks indicate clonotypes detected in ≥2 cells. Statistical testing was not performed due to small sample sizes. (**D**) TRAJ24 (highlighted in the red box) was cloned in two KD patients but not in healthy children. Circos plots showing increased V-J pairs in KD. Left: TRAV-TRAJ pairs; right: TRBV-TRBJ pairs. Only pairs with |Fold Change| > 1 are shown. Enrichment was assessed descriptively; statistical validation was not performed due to the large number of possible V-J combinations and limited sample size. (**E**) Sankey plot of the most frequent αβ V-J pair in KD: TRAV14DV4-J13-TRBV20-1-J2-5. This represents the predominant combination observed; statistical validation was not performed due to limited sample size.

First, we focused on the V and J gene segments, 161 single V and J gene segments shared in KD patients and healthy children, while TRBV6-9 and TRAV 9-1 only appeared in KD patients (Figure 2A upper). TRBV6-9 refers to a particular variable (V) gene segment within the TCR beta chain (TRB) locus, while TRAV9-1 refers to a variable (V) gene segment within the TCR alpha chain (TRA) locus. TRAV26-2, TRAJ35, TRBV30, and TRBJ1-2 on the αβ chains were significantly increased in KD patients compared with healthy children (*p* < 0.05, Fisher’s exact test) (Figure 2C). To analyze whether the top five fragments with the most obvious increase in KD patients were cloned in each sample, clonal fragments were counted, and we found that most V or J gene segments were cloned in at least three individuals (Figure 2D). Furthermore, TRAJ24 was cloned in two KD patients but not in healthy children, suggesting that it was involved in the activation and expansion of T cells in the early stages of KD.

Next, V-J pairing of the α and β chains was analyzed separately. In total, 1732 TRAV-TRAJ pairs of KD patients overlapped with healthy children, and 444 TRAV-TRAJ pairs only appeared in KD patients (*p* < 0.05, hypergeometric test) (Figure 2A down). We then performed a differential analysis based on the frequency of the overlapped TRAV-TRAJ pairs; 48 pairs were significantly increased in KD patients, while 44 pairs were significantly decreased (Figure S1A) (|Fold Change| > 1 and *p* < 0.05, Mann–Whitney U test). TRAJ9, TRAV1-2 and TRAV14DV4 were most involved in the composition of the increased TRAV-TRAJ pairs in KD patients (Figure 2B left). Similar results were obtained in TRBV-TRBJ pairs; 45 pairs significantly increased in KD patients, while 39 pairs were decreased (Figure S1B). TRBJ1-2 and TRBJ1-1 were most involved in the composition of the increased TRBV-TRBJ pairs (Figure 2B, right). These findings are presented as descriptive observations to identify potential V-J pairing patterns associated with KD and warrant further validation in larger cohorts.

Finally, V-J pairs of the αβ chains were compared between KD patients and healthy children, and there were no common αβ VJ pairs. The most frequently used αβ V-J pair in KD patients was TRAV14DV4-J13-TRBV20-1-J2-5 (Figure 2E), while in healthy children, it was TRAV1-2-J33-TRBV20-1-J2-7 (Figure S2).

### 3.3. Preferential Usage of VJ Gene Segments and V-J Pairs in KD Patients Compared with Virus-Infection

**Figure 3:**
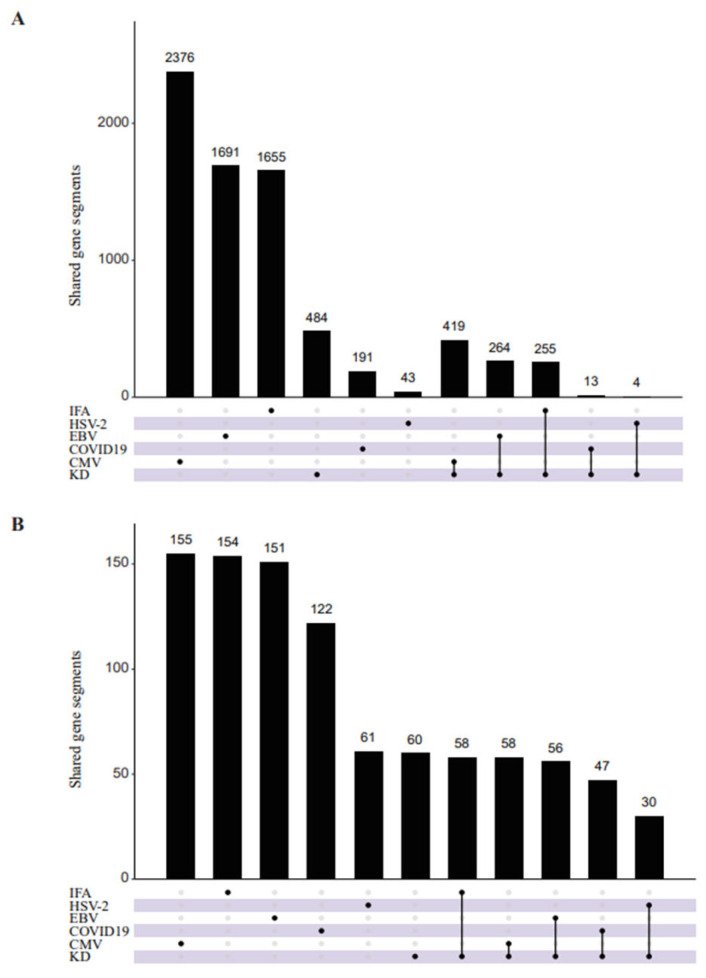

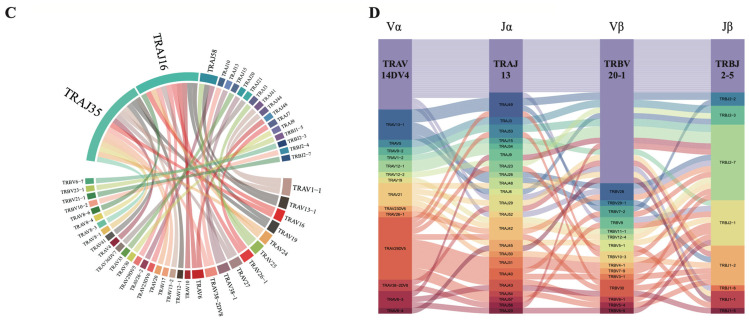
Comparison of VJ gene segments and VJ pairs between KD and virus-infected samples. (**A**) Comparison of V and J gene segments between KD and virus-infected samples. UpSetR plot shows the number of common V and J gene segments. (**B**) Same statistics as in (**A**) for the shared V-J pairing of α and β chains. (**C**) Circos plots show the specific and differential TRAVJ (left) and TRBVJ (right) pairs in KD. (**D**) Sankey plot shows KD-specific αβ-VJ pairs compared with virus-infected samples and healthy children.

To further explore the preferential usage of V and J gene segments in KD patients, we compared the similarities and differences of V and J gene segments between KD patients and the virus-infected samples. The segments of CMV were mostly shared with KD patients, with 419 identical segments (Figure 3A). As for the αβ V-J pairs, the V-J pairs of CMV and IFA samples were mostly shared with KD patients, with 58 identical V-J pairs (Figure 3B). Meanwhile, 60 V-J pairs appeared only in the KD patients (Figure 3B); TRAJ35 and TRAV1-1 were most involved in the composition of significantly different V-J pairs (Figure 3C). The most frequently used αβ V-J pair in KD patients was TRAV14DV4-J13-TRBV20-1-J2-5 compared with virus-infected samples and healthy children (Figure 3D).

### 3.4. CDR3 and Motifs in KD Patients

**Figure 4:**
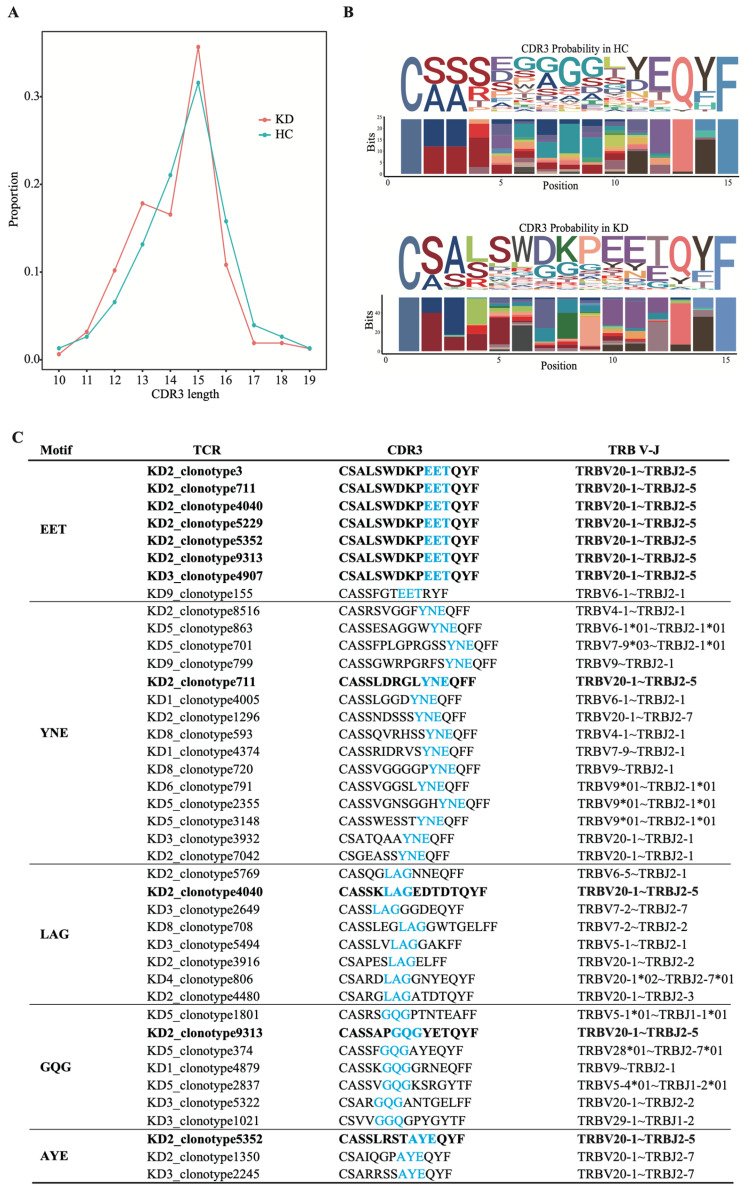
Characteristics of CDR3 in KD. (**A**) Distribution of CDR3 length in KD and healthy children. (**B**) Positional amino acid bias of dominant CDR3β (length = 15) in KD and healthy children. Horizontal axis represents the position of different amino acids. Font sizes for different amino acids represent possibility. (**C**) Enrichment of amino acid motifs in CDR3β of KD. Black fonts highlight the TCRs containing TRAV14DV4-J13-TRBV20-1-J2-5. Sky blue fonts highlight the motif positions in CDR3.

As CDR3 determines the specificity and affinity of antigen recognition, 19 CDR3 amino acid (aa) sequences were analyzed. The distribution of the aa length of CDR3 in KD patients was consistent with healthy children, ranging from 10 aa to 19 aa, and the most common length was 15 aa (Figure 4A). The motifs in healthy children were enriched with uncharged nonpolar amino acids (such as G, L), while the motifs in KD patients were enriched with charged polar amino acids (such as D, K, E) in CDR3βs (Figure 4B).

The frequency of short, contiguous amino acid sequence motifs at defined positions within CDR3 reflects underlying information about the nature of interactions between TCR and pMHC complexes, which determine antigen-specific changes in the TCR repertoire after immune reaction. GLIPH was performed to explore the specific CDR3 motifs in KD patients. Clonally expanded TCRs that appeared in at least three samples in each group were selected for further analysis. In total, 5711 TCRβ KD patients were grouped into 34 groups based on sequence similarity, V-segment bias, CDR3 length bias, and clonal expansion bias. The most frequently utilized pair of motifs in KD patients included EET (Glutamic Acid–Glutamic Acid–Threonine), YNE (Tyrosine–Asparagine–Glutamic Acid), LAG (Leucine–Alanine–Glycine), GQG (Glycine–Glutamine–Glycine), and AYE (Alanine–Tyrosine–Glutamic Acid) (Figure 4C), which reflect the interactions between TCR and peptide–MHC complexes.

## 4. Discussion

Although the etiology of KD remains unclear after nearly half a century of investigation, much evidence suggests that activation of the adaptive (antigen-specific) immune response is related to the pathogenesis of KD [22,23]. A number of circulating regulatory and proinflammatory T cells are altered during the acute phase of KD [23]. Recent advances in the sequencing of TCRs have led to the generation of large datasets of single-chain TCR sequences [24]. Therefore, comparison of the TCR repertoire is essential to explore the association between KD and viral infection.

KD patients shared a lower abundance compared with HSV and COVID-19, suggesting that KD was not activated by a strong specific antigen, which was different from the previous study [25]. T cell receptor diversity is reduced in some diseases, such as in patients with Alzheimer’s clinical syndrome and carcinoma, suggesting weak immunity [26,27]. Here, relatively low diversity was revealed in KD patients compared with healthy children, explaining the progression of KD to some extent.

High phenotypic similarities between KD and MIS-C were discovered, although MIS-C was associated with infection or exposure to SARS-CoV-2, while KD has not been identified as a disease caused by a virus [10]. However, our data showed a high degree of overlap of single-VJ gene segments or V-J pair usage between KD and CMV infection, but the relationship between KD and CMV infection was still not clear. Although we observed overlapping VJ usage between KD and CMV-infected individuals, the absence of shared clonal TCRαβ chains suggests that the immune response to CMV in KD may be restricted or qualitatively distinct from that in typical viral infections.

Analysis of positional motif enrichment within the CDR3 region could help to explore the molecular structure of antigen peptides expressed by MHC molecules on the surface of antigen-presenting cells [28]. Some amino acid motifs were found in TCR containing TRAV14DV4-J13-TRBV20-1-J2-5. This observation raises the possibility that TCR containing TRAV14DV4-J13-TRBV20-1-J2-5 may be involved in a KD-associated immune response, potentially recognizing shared or similar pMHC ligands. However, given the limited sample size and lack of functional validation, the clinical utility of these VJ gene segments and V-J pairs for KD diagnosis remains speculative and requires further investigation in larger cohorts.

Several limitations should be acknowledged. First, the limited sample size may reduce the statistical power of the differential expression analysis. Second, due to the difficulty of retrieving ancestral information from the VDJdb database, the ancestral diversity of participants was not considered. Third, the lack of inclusion of KD patients with varied clinical presentations limited our ability to fully assess the relationship between TCR features and disease progression. Although we identified a TCR motif involving TRAV14DV4-J13-TRBV20-1-J2-5 potentially implicated in KD pathogenesis, confirmatory experiments are still needed. Additionally, the absence of post-treatment follow-up samples precluded longitudinal tracking of viral factors (e.g., CMV) or TCR dynamics, leaving it unclear whether the observed TCR changes are transient or infection-associated. Finally, the TCR repertoire is highly age-dependent, exhibiting limited diversity in infancy and undergoing continuous remodeling during immune maturation. As our analysis mainly relied on public databases without fully accounting for the physiological TCR trajectory in children, potential confounders such as age-matching discrepancies or reference dataset biases may not be completely excluded. Future studies should establish age-stratified pediatric TCR reference profiles to better delineate disease-specific repertoire changes.

## 5. Conclusions

This study provides a preliminary description of TCR repertoire characteristics in KD and exploratory comparisons with viral infections. The TRAV14DV4-J13-TRBV20-1-J2-5 combination was the most frequently observed among KD samples. These findings offer initial insights into potential TCR signatures associated with KD, while the relationship between KD and specific viral infections remains to be further elucidated. As an exploratory analysis, this study provides a foundation for future investigations with larger cohorts and functional assays to clarify the potential role of viral triggers in KD pathogenesis.

## Data Availability

The raw sequence data reported in this paper have been deposited in the Genome Sequence Archive (Genomics, Proteomics & Bioinformatics 2025) at the National Genomics Data Center (Nucleic Acids Res 2025), China National Center for Bioinformation/Beijing Institute of Genomics, Chinese Academy of Sciences (GSA-Human: HRA015796). The data will be made publicly accessible at https://ngdc.cncb.ac.cn/gsa-human (accessed on 28 February 2026), upon publication of this manuscript.

## Supplementary Materials

The following supporting information can be downloaded at: https://www.mdpi.com/article/10.3390/biomedicines14030574/s1, Table S1: Clinical data of the subjects; Table S2: Summary of sequencing quality control metrics for samples. Figure S1: Differential analysis based on the frequency of the overlapped TRAV-TRAJ andTRBV-TRBJ pairs between KD patients and healthy children; Figure S2. The most frequently used αβ V-J pair in healthy children.

## Abbreviations

KD, Kawasaki disease; TCR, T cell receptor; CMV, cytomegalovirus; HSV-2, herpes simplex virus type 2; EBV, Epstein–Barr virus; IFA, influenza A; SARS-CoV-2, severe acute respiratory syndrome coronavirus 2; MIS-C, pediatric multisystemic inflammatory syndrome; CDR3, complementary determining region 3; PBMC, Peripheral Blood Mononuclear Cells; TRA, TCR α chain; TRB, TCR β chain; GLIPH, Grouping of Lymphocyte Interactions by Paratope Hotspots.

## References

[1] McCrindle B.W., Rowley A.H., Newburger J.W., Burns J.C., Bolger A.F., Gewitz M., Baker A.L., Jackson M.A., Takahashi M., Shah P.B. (2017). Diagnosis, Treatment, and Long-Term Management of Kawasaki Disease: A Scientific Statement for Health Professionals From the American Heart Association. Circulation.

[2] Mahmoudinezhad Dezfouli S.M., Salehi S., Khosravi S. (2022). Pathogenic and therapeutic roles of cytokines in Kawasaki diseases. Clin. Chim. Acta.

[3] Makino N., Nakamura Y., Yashiro M., Kosami K., Matsubara Y., Ae R., Aoyama Y., Yanagawa H. (2019). Nationwide epidemiologic survey of Kawasaki disease in Japan, 2015-2016. Pediatr. Int..

[4] Rife E., Gedalia A. (2020). Kawasaki Disease: An Update. Curr. Rheumatol. Rep..

[5] Rosenfeld N., Tasher D., Ovadia A., Abiri S., Dalal I. (2020). Kawasaki disease with a concomitant primary Epstein—Barr virus infection. Pediatr. Rheumatol. Online J..

[6] Usta Guc B., Cengiz N., Yildirim S.V., Uslu Y. (2008). Cytomegalovirus infection in a patient with atypical Kawasaki disease. Rheumatol. Int..

[7] Lim J.H., Kim Y.K., Min S.H., Kim S.W., Lee Y.H., Lee J.M. (2021). Seasonal Trends of Viral Prevalence and Incidence of Kawasaki Disease: A Korea Public Health Data Analysis. J. Clin. Med..

[8] Hagiwara K., Komura H., Kishi F., Kaji T., Yoshida T. (1992). Isolation of human herpesvirus-6 from an infant with Kawasaki disease. Eur. J. Pediatr..

[9] Verdoni L., Mazza A., Gervasoni A., Martelli L., Ruggeri M., Ciuffreda M., Bonanomi E., D’Antiga L. (2020). An outbreak of severe Kawasaki-like disease at the Italian epicentre of the SARS-CoV-2 epidemic: An observational cohort study. Lancet.

[10] Sharma C., Ganigara M., Galeotti C., Burns J., Berganza F.M., Hayes D.A., Singh-Grewal D., Bharath S., Sajjan S., Bayry J. (2021). Multisystem inflammatory syndrome in children and Kawasaki disease: A critical comparison. Nat. Rev. Rheumatol..

[11] Xie Z., Huang Y., Li X., Lun Y., Li X., He Y., Wu S., Wang S., Sun J., Zhang J. (2022). Atlas of circulating immune cells in Kawasaki disease. Int. Immunopharmacol..

[12] Greene E., MacIver N.J. (2022). Targeting T cell (oxidative) metabolism to improve immunity to viral infection in the context of obesity. Front. Immunol..

[13] Ye Q., Wang D.J., Lan B., Mao J.H. (2023). T-cell and B-cell repertoire diversity are selectively skewed in children with idiopathic nephrotic syndrome revealed by high-throughput sequencing. World J. Pediatr..

[14] Weng N.P. (2023). Numbers and odds: TCR repertoire size and its age changes impacting on T cell functions. Semin. Immunol..

[15] Heather J.M., Spindler M.J., Alonso M.H., Shui Y.I., Millar D.G., Johnson D.S., Cobbold M., Hata A.N. (2022). Stitchr: Stitching coding TCR nucleotide sequences from V/J/CDR3 information. Nucleic Acids Res..

[16] Dionne A., Meloche-Dumas L., Desjardins L., Turgeon J., Saint-Cyr C., Autmizguine J., Spigelblatt L., Fournier A., Dahdah N. (2017). N-terminal pro-B-type natriuretic peptide diagnostic algorithm versus American Heart Association algorithm for Kawasaki disease. Pediatr. Int..

[17] Wang P., Jin X., Zhou W., Luo M., Xu Z., Xu C., Li Y., Ma K., Cao H., Huang Y. (2021). Comprehensive analysis of TCR repertoire in COVID-19 using single cell sequencing. Genomics.

[18] Huang H., Wang C., Rubelt F., Scriba T.J., Davis M.M. (2020). Analyzing the Mycobacterium tuberculosis immune response by T-cell receptor clustering with GLIPH2 and genome-wide antigen screening. Nat. Biotechnol..

[19] Zhang S., Chen X., Jin E., Wang A., Chen T., Zhang X., Zhu J., Dong L., Sun Y., Yu C. (2025). The GSA Family in 2025: A Broadened Sharing Platform for Multi-omics and Multimodal Data. Genom. Proteom. Bioinform..

[20] (2025). CNCB-NGDC Members and Partners. Database Resources of the National Genomics Data Center, China National Center for Bioinformation in 2025. Nucleic Acids Res..

[21] Dupic T., Marcou Q., Walczak A.M., Mora T. (2019). Genesis of the alphabeta T-cell receptor. PLoS Comput. Biol..

[22] Wang N., Chen Z., Zhang F., Zhang Q., Sun L., Lv H., Wang B., Shen J., Zhou X., Chen F. (2022). Intravenous Immunoglobulin Therapy Restores the Quantity and Phenotype of Circulating Dendritic Cells and CD4(+) T Cells in Children With Acute Kawasaki Disease. Front. Immunol..

[23] Wang Z., Xie L., Ding G., Song S., Chen L., Li G., Xia M., Han D., Zheng Y., Liu J. (2021). Single-cell RNA sequencing of peripheral blood mononuclear cells from acute Kawasaki disease patients. Nat. Commun..

[24] Shugay M., Bagaev D.V., Zvyagin I.V., Vroomans R.M., Crawford J.C., Dolton G., Komech E.A., Sycheva A.L., Koneva A.E., Egorov E.S. (2018). VDJdb: A curated database of T-cell receptor sequences with known antigen specificity. Nucleic Acids Res..

[25] Rowley A.H., Arrollo D., Shulman S.T., Torres A., O’Brien A., Wylie K., Kim K.A., Baker S.C. (2023). Analysis of Plasmablasts From Children With Kawasaki Disease Reveals Evidence of a Convergent Antibody Response to a Specific Protein Epitope. J. Infect. Dis..

[26] Qian L., Zhaohui Z., Yaping X., Zhentian L., Zhentao L., Qiqi W., Yangchun G., Yan’e L., Wencheng Y., Fumei Y. (2021). Blood T cell diversity associated with the prognosis of advanced non-small cell lung carcinoma treated with first-line pemetrexed based chemotherapy. Thorac. Cancer.

[27] Joshi C., Sivaprakasam K., Christley S., Ireland S., Rivas J., Zhang W., Sader D., Logan R., Lambracht-Washington D., Rosenberg R. (2022). CSF-Derived CD4(+) T-Cell Diversity Is Reduced in Patients With Alzheimer Clinical Syndrome. Neurol. Neuroimmunol. Neuroinflamm..

[28] Brenke R., Hall D.R., Chuang G.Y., Comeau S.R., Bohnuud T., Beglov D., Schueler-Furman O., Vajda S., Kozakov D. (2012). Application of asymmetric statistical potentials to antibody-protein docking. Bioinformatics.

