# Supplementary material for: TCR Repertoire Analysis Unveils the Link Between Kawasaki Disease and Viral Infection
Source: Biomedicines. 2026 Mar 3;14(3):574. doi: 10.3390/biomedicines14030574 (PMC13024496; doi:10.3390/biomedicines14030574)
Supplement: Supplementary file 1 [file biomedicines-14-00574-s001.zip › Figure Legend for Supplementary Figures.pdf]

Figure S1. Differential analysis based on the frequency of the overlapped TRAV-TRAJ andTRBV-TRBJ pairs between KD patients and healthy children.

Figure S2. The most frequently used αβ V-J pair in healthy children
