# Supplementary material for: TCR Repertoire Analysis Unveils the Link Between Kawasaki Disease and Viral Infection
Source: Biomedicines. 2026 Mar 3;14(3):574. doi: 10.3390/biomedicines14030574 (PMC13024496; doi:10.3390/biomedicines14030574)
Supplement: Supplementary file 1 [file biomedicines-14-00574-s001.zip › Supplementary Table S1.pdf]

Supplementary Table S1. Clinical data of the subjects

		Sex	Age	Clinical findings	Laboratory findings
Healthy Children (HC)	HC1	Female	1y5m	N/A	N/A
	HC2	male	1y8m	N/A	N/A
	HC3	male	1y8m	N/A	N/A
KD patients	KD1	male	1y2m	Fever for 5 days;<br>Erythema of lips, strawberry tongue;<br>Bilateral bulbar conjunctival injection without exudate;<br>Erythema and edema of the hands and feet;<br>Cervical lymphadenopathy (1.6 cm diameter);	WBC: 9.5 × 10^9;<br>Neutrophil ratio: 64.2%;<br>CRP: 61.32mg/L;<br>ESR: 28mm/h;<br>IL-6: 22.3pg/ml;<br>IL-10: 12.5pg/ml;
	KD2	male	1y3m	Fever for 5 days;<br>Erythema of lips, strawberry tongue;<br>Bilateral bulbar conjunctival injection without exudate;<br>Cervical lymphadenopathy (1.6 cm diameter);	WBC: 19.65 × 10^9;<br>Neutrophil ratio: 73.6%;<br>CRP: 86.70mg/L;<br>ESR: 50mm/h;<br>IL-6: 51.1pg/ml;<br>IL-10: 16.0pg/ml;
	KD3	male	1y5m	Fever for 5 days;<br>Erythema of lips, strawberry tongue;<br>Bilateral bulbar conjunctival injection without exudate;<br>Cervical lymphadenopathy (1.7 cm diameter);	WBC: 11.54 × 10^9;<br>Neutrophil ratio: 51.1%;<br>CRP: 61.84mg/L;<br>ESR: 36mm/h;<br>IL-6: 73.7pg/ml;<br>IL-10: 25.4pg/ml;

WBC indicates White blood cell count; CRP, C-reactive protein; ESR, Erythrocyte sedimentation rate; IL, Interleukin.
