# Supplementary material for: TCR Repertoire Analysis Unveils the Link Between Kawasaki Disease and Viral Infection
Source: Biomedicines. 2026 Mar 3;14(3):574. doi: 10.3390/biomedicines14030574 (PMC13024496; doi:10.3390/biomedicines14030574)
Supplement: Supplementary file 1 [file biomedicines-14-00574-s001.zip › Supplementary Table S2.pdf]

Supplementary Table S2. Summary of sequencing quality control metrics for samples

Metric	HC-1	HC-2	HC-3	KD-1	KD-2	KD-3
Estimated Number of Cells	3,339	5,686	2,532	6,099	10,066	6,053
Mean Read Pairs per Cell	6,835	3,945	8,726	3,862	2,214	4,270
Mean Used Read Pairs per Cell	3,784	2,600	4,110	2,674	1,386	3,030
Fraction Reads in Cells	65.50%	76.50%	56.90%	82.00%	76.10%	82.50%
Reads Mapped to Any V(D)J Gene	79.40%	82.80%	78.00%	81.70%	79.20%	84.10%
Reads Mapped to TRA	15.60%	21.10%	13.30%	18.80%	17.50%	21.30%
Reads Mapped to TRB	63.70%	61.60%	64.60%	62.70%	61.60%	62.70%
Median TRA UMIs per Cell	1	1	0	1	1	1
Median TRB UMIs per Cell	4	4	4	6	5	5
Paired Clonotype Diversity	909.03	2927.11	251.85	1025.33	1554.68	862.31
Cells With TRA Contig	57.20%	67.30%	41.40%	67.70%	60.70%	71.20%
Cells With TRB Contig	97.60%	98.20%	98.60%	98.70%	98.20%	98.30%
Cells With CDR3-annotated TRA Contig	52.70%	61.80%	36.60%	63.20%	55.90%	66.80%
Cells With CDR3-annotated TRB Contig	96.70%	97.30%	98.30%	98.00%	97.50%	97.60%
Cells With V-J Spanning TRA Contig	54.40%	63.90%	39.20%	64.50%	57.20%	67.70%
Cells With V-J Spanning TRB Contig	96.60%	97.00%	98.20%	97.90%	97.30%	97.40%
Cells With Productive TRA Contig	45.80%	55.50%	31.00%	57.40%	49.80%	60.00%
Cells With Productive TRB Contig	96.00%	96.30%	98.00%	97.30%	96.70%	96.90%
Number of Cells With Productive V-J Spanning Pair	1,398	2,945	735	3,340	4,678	3,445
Cells With Productive V-J Spanning Pair	41.90%	51.80%	29.00%	54.80%	46.50%	56.90%
Cells With Productive V-J Spanning (TRA, TRB) Pair	41.90%	51.80%	29.00%	54.80%	46.50%	56.90%

GLIPH parameters (except for --tcr and --refdb, which were specified as the input CDR3 sequences and reference database, all other parameters were set to default values)

Usage: /Bio/Bin/pipeline/GD_trustVDJ/dev/gliph/gliph-group-discovery.pl
--tcr=seqs.txt Tab-separated input with column 1 containing CDR3s
--refdb=vdfasta.fa Optional alternative reference database
--gccutoff=1 Global coverage distance cutoff, or global convergence simulation file. Calculated at runtime if not specified.
--motif_file=motif.txt Optional local convergence significant motif list (calculated if not specified)
--simdepth=1000 Simulated resampling depth for non-parametric convergence significance tests
--lcmimp=0.01 Local convergence minimum p-value for significance (0.01 by default)
--lcmmove=10 Local convergence minimum observed vs expected fold change (10 by default)
--kmer_mindepth=3 Minimum observations of kmer for it to be evaluated
--global=1 Search for global TCR similarity (Default 1)
--local=1 Search for local TCR similarity (Default 1)
--make_depth_fig=0 Perform repeat random samplings at the test set depth in order to visualize convergence
--discontinuous=0 Allow discontinuous motifs (Default 0)
--positional_motifs=0 Restrict motif clustering to a shared position that is fixed from the N-terminal end of CDR3
--cdr3len_stratify=0 Stratify by shared CDR3 length distribution (Default 0)
--vgene_constrain=0 Stratify by shared V-gene frequency distribution (Default 0)
--public_tcrs=0 Reward motifs in public TCRs (Default 0)
--structboundaries=1 Use structural boundaries (Default 1)
--length_stratify=1 Apply length stratification to match CDR3 length distribution to control set random samplings (default 0)
