# Supplementary figures and images for: TCR Repertoire Analysis Unveils the Link Between Kawasaki Disease and Viral Infection
Source: Biomedicines. 2026 Mar 3;14(3):574. doi: 10.3390/biomedicines14030574 (PMC13024496; doi:10.3390/biomedicines14030574)

**A**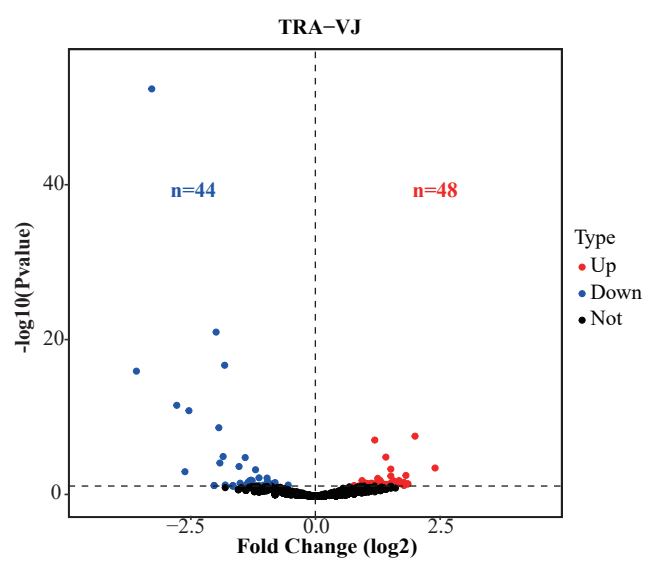**B**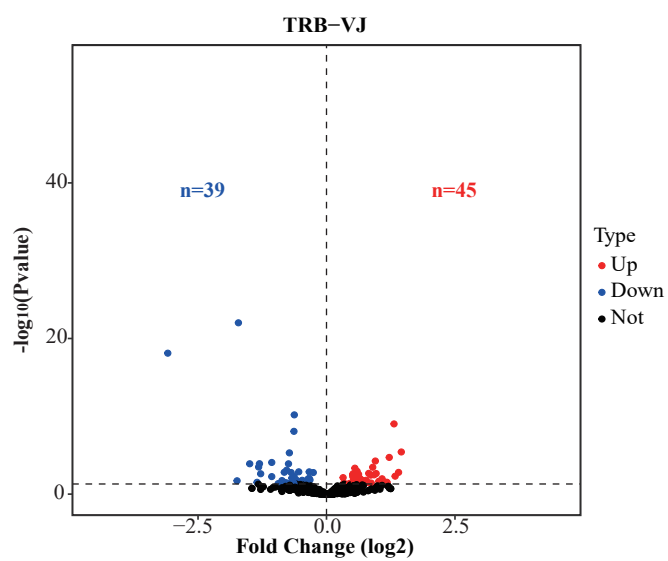

Supplement: Supplementary file 1 [file biomedicines-14-00574-s001.zip › Supplementary Figure S1.pdf]

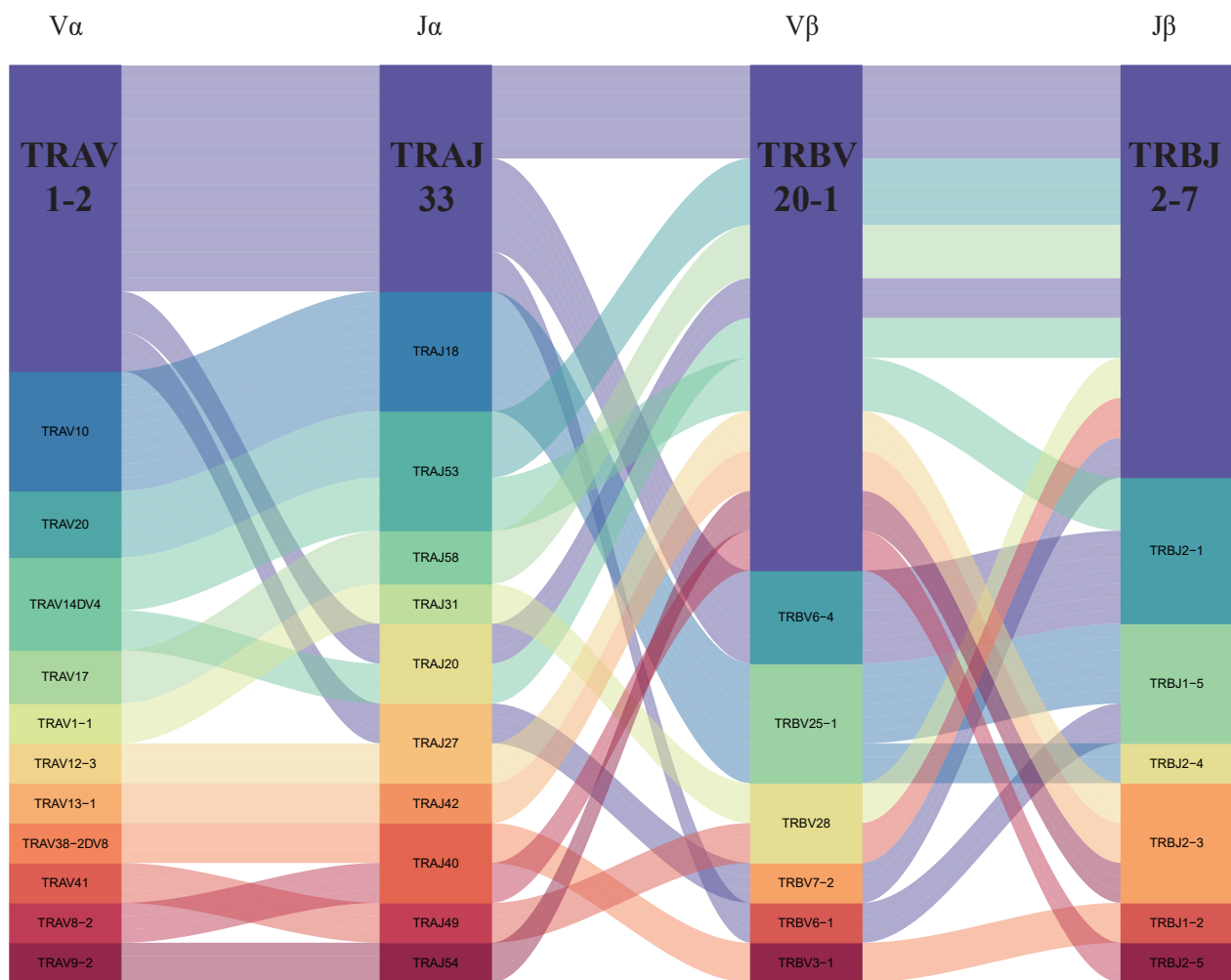

Supplement: Supplementary file 1 [file biomedicines-14-00574-s001.zip › Supplementary Figure S2.pdf]
